# Atomistic and experimental study on thermal conductivity of bulk and porous cerium dioxide

**DOI:** 10.1038/s41598-019-42807-5

**Published:** 2019-04-19

**Authors:** Linu Malakkal, Anil Prasad, Dotun Oladimeji, Ericmoore Jossou, Jayangani Ranasinghe, Barbara Szpunar, Lukas Bichler, Jerzy Szpunar

**Affiliations:** 1 Department of Mechanical Engineering, University of Saskatchewan, Saskatoon, Canada; 2 School of Engineering University of British Columbia-Okanagan Kelowna, Kelowna, Canada; 3 Department of Physics and Engineering Physics, University of Saskatchewan, Saskatoon, Canada

**Keywords:** Materials for energy and catalysis, Ceramics

## Abstract

Cerium dioxide (CeO_2_) is a surrogate material for traditional nuclear fuels and an essential material for a wide variety of industrial applications both in its bulk and nanometer length scale. Despite this fact, the underlying physics of thermal conductivity (*k*_*L*_), a crucial design parameter in industrial applications, has not received enough attention. In this article, a systematic investigation of the phonon transport properties was performed using *ab initio* calculations unified with the Boltzmann transport equation. An extensive examination of the phonon mode contribution, available three-phonon scattering phase space, mode Grüneisen parameter and mean free path (MFP) distributions were also conducted. To further augment theoretical predictions of the *k*_*L*_, measurements were made on specimens prepared by spark plasma sintering using the laser flash technique. Since the sample porosity plays a vital role in the value of measured *k*_*L*_, the effect of porosity on *k*_*L*_ by molecular dynamics (MD) simulations were investigated. Finally, we also determined the nanostructuring effect on the thermal properties of CeO_2_. Since CeO_2_ films find application in various industries, the dependence of thickness on the in-plane and cross-plane *k*_*L*_ for an infinite CeO_2_ thin film was also reported.

## Introduction

The lattice thermal conductivity (*k*_*L*_) depends on the mean free path (MFP), i.e. the distance travelled by the phonons before the occurrence of any scattering events. A detailed quantitative understanding of the mode dependent phonon properties and the bulk MFPs in each material is imperative to exercise control over its thermal management. Furthermore, the thermal transport properties of the bulk and the nanostructured (wires, films, porous, and nanocrystalline material) counterparts of a given material differ significantly due to increased boundary scattering in the latter. Understanding this phenomenon becomes necessary when studying thermal conductivity of materials such as Cerium dioxide (CeO_2_), which finds applications in both bulk and nanostructured form. Bulk CeO_2_ is frequently used as a surrogate material in the nuclear industry, as an oxygen storage material in automobile exhaust systems^1^, and as an electrolyte in solid oxide fuel cells^2^. Also, nanostructured CeO_2_ is used for diverse applications in various industries. For instance, in electronics, the thin films of CeO_2_ are among the most prospective buffer layers for high-temperature semiconductor and ferroelectric films deposited on silicon^3,4^. In the nuclear industry, CeO_2_ thin film deposits have been proposed for controlling the oxidation of nickel alloys^5^, and microspheres of CeO_2_ have been synthesized for space nuclear applications^6^. Unfortunately, to date, accurate atomic-scale investigation on the details of mode-wise thermal transport properties and MFP of CeO_2_ have not been reported.

Although several studies have reported the *k*_*L*_ of CeO_2_, the value remains ambiguous. Experimentally, *k*_*L*_ of CeO_2_ has been determined using laser flash technique. Nelson *et al*.^7^ and Khafizov *et al*.^8^ measured the *k*_*L*_ of 95% dense CeO_2_ pellet and reported respective values of 6.6 W/mK and 17.5 W/mK (correcting for porosity using a modified Loeb expression), at room temperature. The *ab initio* theoretical calculations for the *k*_*L*_ of CeO_2_ performed by Xiao *et al*.^9^ and the molecular dynamics (MD) study by Khafizov *et al*.^8^ predict the *k*_*L*_ as 12 W/mK and 19 W/mK respectively. However, Xiao *et al*.^9^ used the analytical Slack model^10^, which heavily relied on the fitting parameters that may obscure the underlying physics of heat transfer. Whereas, the MD simulations performed by Khafizov *et al*.^8^ could have over-predicted the *k*_*L*_ at lower temperatures as MD simulations do not account for intrinsic scattering at these temperatures. Right through the literature, the value of *k*_*L*_ of CeO_2_ at room temperature has varied between (6.6 W/mK and 19 W/mK) and thus exists a disparity. To resolve this aberration, we perform an *ab initio* calculation using density functional theory (DFT)^11^, density functional perturbation theory (DFPT)^12^ and lattice dynamics in concord with the Boltzmann transport equation (BTE) using ShengBTE^13^. This theoretical approach does not require any assumption on the phonon lifetime to predict the *k*_*L*_. BTE has been successfully utilized in the investigations of phonon transport properties for many materials^14–19^ with high accuracy. In addition to the *ab initio* calculations, MD simulations were carried out to understand the effect of porosity on the *k*_*L*_ of CeO_2_. Moreover, using the laser flash experiment technique, the thermal conductivity of porous CeO_2_ pellets prepared by spark plasma sintering (SPS) method has also been reported be a restrictive factor for the heat transfer.

In nanostructured materials, where the phonon MFP is comparable to the grain size, grain boundaries can modulated thermoreflectance technique and an analytical solution of BTE to understand the thermal transport properties of ceria thin films grown by unbalanced magnetron sputtering. The significantly reduced conductivity of these thin films compared to the bulk CeO_2_ was attributed to the combined effect of the point defects, grain boundaries, and dislocations. However, their study was not able to shed light on the influence of nanostructuring. For nanoscale applications, the characteristic lengths of the nanostructures can be a limiting factor in the thermal transport. To have a comprehensive understanding of this limiting factor, it is critical to know MFP of CeO_2_. It is also important to know what specific length of the nanostructure is going to be significant for CeO_2_. Therefore, in this work, the *ab initio* prediction of the MFP, the relaxation time of the phonons, their mode-wise contribution to thermal conductivity, and the effect of nanostructuring on the reduction of thermal conductivity were investigated. These findings will aid the selection of the size and thickness of the nanostructures in tuning the thermal properties. Furthermore, the effect of nanostructuring by studying the cross plane and in-plane thermal conductivity of CeO_2_ thin films and the impact of the thickness of the thin films and temperature on the thermal conductivity was also performed. These predicted results not only enable the accurate explanation of the experimental results but also guide further designs and applications.

## Results and Discussion

### Crystal Structure and elastic constants

**Figure 1:**
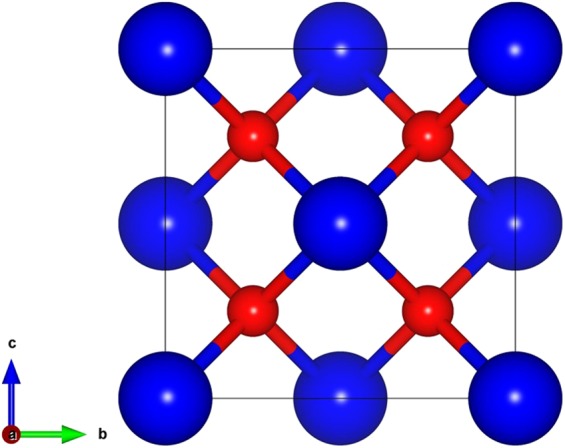
Crystal structure of CeO_2_ in the conventional cubic unit cell (blue and red represent Ce and O atoms respectively.

CeO_2_ has a fluorite structure with three independent atoms per unit cell and belongs to the space group of Fm-3m (225), as shown in Fig. 1. The equilibrium lattice constants were obtained by minimizing the total energy with respect to the lattice parameter and atomic positions. The detailed description of the optimized parameter and the pseudopotential used for the simulation were provided in section 4.1. Table 1. presents the calculated lattice constant (*a*), bulk modulus (*B*) and stiffness constants (*C*_*ij*_) of CeO_2_ in comparison with the values from the previous DFT calculations^20–23^ and the experiment^24^. The PBEsol functional predicted the lattice constant within an error less than 0.01% compared with the experimental data reported by Nakajima *et al*.^24^ As expected, the new PBEsol functional was able to quantify the ground state structural properties more accurately than previous DFT calculations performed using linear density approximation (LDA)^20^, GGA^21^, Hartwigsen- Goedecker-Hutter (HGH)^22^ and PAW^23^ pseudopotentials.

**Table 1:** Structural and mechanical properties of CeO_2_ compared with previous DFT calculations and the experiment.

*a* (Å)	*B* (GPa)	*C*_11_ (GPa)	*C*_12_ (GPa)	*C*_44_ (GPa)	*G* (GPa)	*Y* (GPa)	*η*	References
5.409	192	355	110	62	82	215	0.313	This work (PBEsol)
5.37	203	371	117	68	—	—	—	LDA^20^
5.45	193	—	—	—	—	—	—	PAW^23^
5.37	210	386	124	73	—	—	—	HGH^22^
5.44	181	347	97	51	74	195	0.218	GGA^21^
5.41	204	403	105	60	—	—	—	Exp.^24^

The elastic constants are important parameters that provide information on the properties of a material such as stiffness, strength, mechanical stability, hardness, and ductility or brittleness^25^. We evaluated the single crystal stiffness constants of CeO_2_ by using a stress-strain method^26,27^ with the help of our in-house interface qe-nipy-advanced^28^. The stiffness constants for a mechanically stable cubic structure should satisfy the following Born’s mechanical stability criteria^29^ ${C}_{11}-{C}_{12} > 0,\,{C}_{11}+2{C}_{12} > 0,\,{C}_{44} > 0$. The listed stiffness constants have satisfied these criteria indicating that the system is mechanically stable.

From the calculated stiffness constants, the polycrystalline bulk modulus, shear modulus, Young’s modulus and Poisson’s ratio (listed in Table 1) were determined using the Voigt-Reuss-Hill approach^30–32^. From Table 1. the bulk modulus values (192 GPa) at zero temperature obtained from PBEsol method is only about 6% lower than the experimental value of 204 GPa^24^ at room temperature. Since the calculated structural and mechanical properties are in excellent agreement with the experimental data, the same PBEsol functional pseudopotentials were used for the study of phonon properties and the *k*_*L*_ of CeO_2_.

### Lattice dynamics

**Figure 2:**
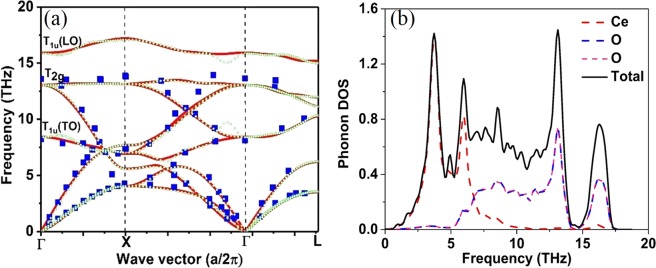
(**a**) Phonon spectra of CeO_2_ predicted by the linear response approach using q-point of 6 × 6 × 6 (red line) and the finite displacement (FD) methods (dotted green line) compared with the experiment done by Clausen K. *et al*.^37^ (blue dots). (**b**) Partial (dotted lines) and total phonon density of states (solid black line).

Lattice dynamics is critical for understanding the thermal properties of crystalline solids at finite temperatures. For instance, the phonon densities of states are required to evaluate thermodynamic properties such as thermal expansion coefficient (*α*), *C*_*V*_, entropy (*S*) and *k*_*L*_. Moreover, the fundamental reasons for unique thermal characteristics of a material can be ascertained by analyzing the phonon scattering mechanism through phonon group velocities, and phonon mean free path, relaxation time and phonon scattering phase space^33^. At present, two approaches the linear-response approach^34^ and the direct approach^35,36^ are widely used to evaluate the phonon dispersion. In Fig. 2a the calculated phonon dispersion spectra using these two approaches the linear-response approach (QE) and the direct approach (Phonopy) are presented along the Γ-X-Γ-L high symmetry points in the Brillouin zone and compared with the known experimental data.

The predicted phonon spectra using both the linear-response approach and the finite displacement (FD) methods are in good agreement with the experimental data generated by Clausen K *et al*.^37^, for the entire Brillouin zone using inelastic neutron scattering at 293 K. CeO_2_ has three (n) atoms per primitive unit cell and thus there are in three dimensions (d = 3) three acoustic mode phonons and d (n − 1) = 6 optical phonons. The group theory analysis gives the decomposition of the zone centre modes as doubly degenerate transverse optical (TO) mode, the triply degenerate Raman active mode and the non-degenerate longitudinal optical (LO) mode. The three-zone center frequencies are 8.42 (8.15), 13.03 (13.94), and 15.93(17.84) THz respectively (shown in brackets are the experimental value from the ref.^38^). The partial lifting of degeneracy between the LO and TO phonons at the Brillouin zone centre is due to the polarization effect, which indicates that the CeO_2_ is a polar material as shown in Fig. 2a The Born effective charge for an insulator is a measure of the change in electronic polarization due to ionic displacements. These charges are essential for elucidating the physical understanding of piezoelectric and ferroelectric properties since they describe the coupling between lattice displacements and the electric field. The Born effective charges, ${Z}_{Ce}^{\ast }$ = 5.793 (5.746) ${Z}_{O}^{\ast }$ = 2.896 (2.873), agrees well with the values reported (shown in brackets) by Xiao *et al*.^9^ The calculated dielectric constant value 7.87 (6.0) is in reasonable agreement the work done by Santha *et al*.^38^.

The partial and total phonon density of states is shown in Fig. 2b. The partial density of the state shows that the frequency vibrations lower than 6.0 THz are dominated by the Ce ions and the higher frequency vibrations are mainly contributed by the dynamics of O ions. The phonon dispersion of CeO_2_ along Γ-X-Γ-L, high symmetry points in the Brillouin zone does not display frequency gap between optical and acoustic phonons. The absence of frequency gap in case of CeO_2_ indicates that three-phonon scattering is dominant resulting in relatively lower phonon relaxation time thereby leading to lower *k*_*L*_. The accurate prediction of phonon dispersion curve of CeO_2_ using both the linear-response approach and direct approach enables reasonable precision in the projection of phonon-assisted properties.

### Three-phonon scattering phase space and Grüneisen parameter

**Figure 3:**
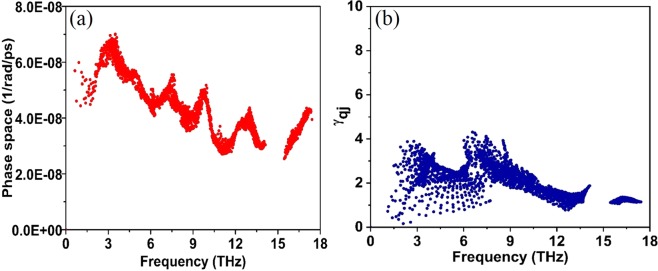
(**a**) Three-phonon scattering phase space. (**b**) Mode Grüneisen parameter (γ_qj_).

Three-phonon scattering phase space (*P*_3_) quantitatively describes the number of scattering channels available for a phonon being scattered^39^. The available *P*_3_ gives an insight into the *k*_*L*_ of a material i.e. larger available *P*_3_, will have more channels for scattering and subsequently lower *k*_*L*_. The *P*_3_ is solely determined from a material’s phonon spectra and is defined as^33^,

1 $${P}_{3}(q,j)=\frac{1}{3{\rm{\Omega }}}(2{P}_{3}^{(+)}(qj)+{P}_{3}^{(-)}(qj))$$

where *q* is the momenta of phonons, *j* is the phonon branches, ${\rm{\Omega }}={n}_{j}^{3}{V}_{BZ}^{2}$ is the normalized factor equal to unrestricted phase space for each type of process. ${P}_{3}^{+}$ and ${P}_{3}^{-}$ are the absorption and emission processes respectively, defined by,

2 $${P}_{3}^{\pm }(q,j)=\frac{1}{{N}_{q}}\sum _{{q}_{1},{q}_{2},{j}_{1},{j}_{2}}\,\delta ({\omega }_{qj}\pm {\omega }_{{q}_{1}{j}_{1}}-{\omega }_{{q}_{2}{J}_{2}}){\delta }_{q\pm {q}_{1},q+G}$$

where *N*_*q*_ is the total number of *q* points in the first Brillouin zone. Figure 3a shows the *P*_3_ of CeO_2_ for the acoustic and optical modes. To gain a quantitative understanding of available *P*_3_ on *k*_*L*_, the total volume in phase space for three phonon processes (*P*_3_*_total*) for CeO_2_ is compared with our previously reported results of a relatively higher *k*_*L*_ material such as silicon carbide (420 W/mK at 300 K)^17^. The *P*_3_*_total* (in units of 1/rad/ps) predicted along the same Brillioun path is higher for CeO_2_ (4.18 × 10^−3^) than SiC (1.66 × 10^−3^); clearly indicating that CeO_2_ has a larger three phonons scattering phase space, more channels for scattering and hence lower thermal conductivity than SiC. Lindsay and Broido^33^ have already established the phase space available for the three-phonon scattering for semiconductors. In this work, the possible *P*_3_ for a lanthanide oxide was illustrated, and these findings would aid in qualitatively determining the thermal conductivities of new materials with known phonon dispersion.

Grüneisen parameter (*y*) illustrates the anharmonicity in a crystal and is defined as the shift in phonon frequency with change in volume. It is an established fact that anharmonicity in an ordered crystal structure determines the strength of each scattering channel and the efficiency of each phonon mode in heat conduction is inversely proportional to square of Grüneisen parameter. To quantitatively examine the anharmonicity of CeO_2_, we plot the mode Grüneisen parameters at zero temperature (as shown in Fig. 3b) derived from the third-order anharmonic force constants (shown in Eq. (3)) as implemented in the ShengBTE^13^ and is defined as:

3 $${\gamma }_{p}(q){|}_{anh}=-\,\frac{1}{6{\omega }_{p}{(q)}^{2}}\sum _{nl^{\prime} }\sum _{n^{\prime\prime} l^{\prime\prime} }\sum _{\alpha \beta \gamma }{\Phi }_{{n}^{o},n^{\prime} l^{\prime} ,n^{\prime\prime} {\rm{l}}^{\prime\prime} }^{\alpha \beta \gamma }\times (\frac{\,{e}_{{\alpha }_{\eta }}^{p}({q}^{\ast }){e}_{\beta \eta ^{\prime} }^{p}(q)}{\sqrt{{M}_{\eta }{M^{\prime} }_{\eta }}}){e}^{-iq{R^{\prime} }_{i}{r}_{\eta ^{\prime\prime} l^{\prime\prime} \gamma ^{\prime} }}$$

where ${{\rm{\Phi }}}_{{n}^{o},n^{\prime} l^{\prime} ,nl}^{\alpha \beta \gamma }$ *a*re the third-order force constants, *e* is the phonon eigenvectors, *n* denotes the *n*^*th*^ primitive cell in the supercell, *α* is the Cartesian components of *x*, *y*, or, *z*, *M*_*n*_ refers to the mass of the atomic type *n* in the primitive cell and *r*_*ηl*_ is the position vector of the *η*^*th*^ atom in the *l*^*th*^ primitive cell. According to Fig. 3b, the sum of all mode Grüneisen parameters are positive indicating volume expansion. At zero temperature, the average Grüneisen coefficient over the whole frequency range was 2.07. The mode Grüneisen parameter near 7 THz (near the TO mode) is higher, indicating that the scattering of acoustic phonons by the optical mode caused hindrance to heat conduction. Changes in mode Grüneisen parameter with different q-points in the second order force constant and the neighboring atoms in the third order force constant is given as Supplementary Information (S.I) in S.I.1 (Fig. S1).

### Theoretical prediction of thermal conductivity and mode contribution

**Figure 4:**
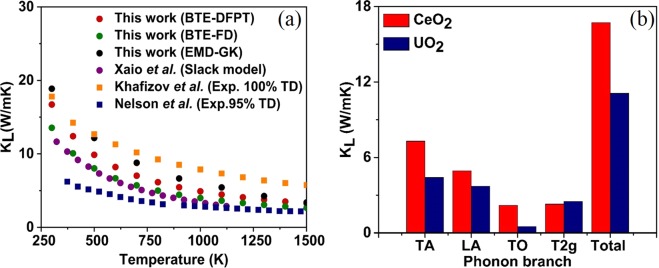
(**a**) The temperature dependence of the phonon *k*_*L*_ in CeO_2_ using BTE and experiment compared to previous theoretical^9^ and experimental^7^ values of *k*_*L*_. (**b**) The mode-wise *k*_*L*_ of CeO_2_ in comparison with the simulated results for UO_2_ by Pang *et al*.^42^.

The full iterative solution of BTE for CeO_2_ is shown in Fig. 4a. As expected, the *k*_*L*_ of CeO_2_ decreases with increasing temperature because the phonon-phonon scattering dominates the *k*_*L*_ at high temperatures. The *k*_*L*_ at 300 K is 16.71 W/mK. Our predictions were made assuming a defect-free crystal and thus could potentially represent the upper limit of *k*_*L*_. The previous experimental value of thermal conductivity for CeO_2_ appears to be scattered. For example, for ~95% dense pellets at 373 K, Jakub *et al*.^40^ and Nelson *et al*.^7^ reported a value of 9.58 W/mK and 6.2 W/mK, respectively. These scattered values of *k*_*L*_ reported in the literature are generally justified by pointing to microstructures. This may not be true under all circumstances. In these situations, an accurate theoretical prediction without the usage of fitting parameters can significantly help in arriving at a meaningful physical explanation. The highest reported experimental value for CeO_2_ for a ~95% dense pellet at room temperature is by Jakub *et al*.^40^ (14.1 W/mK) and our theoretical predictions at 300 K is 16.71 W/mK (red dots) for 100% dense single crystal, are in excellent agreement. Furthermore, the difference in *k*_*L*_ values obtained through our theoretical prediction and the previously reported simulated value of 12 W/mK at 300 K by Xiao *et al*.^9^ (using fitting parameters) clearly indicate the apparent disadvantages of using fitting parameters. We also noted that Xiao *et al*. obtained the second order force constant required for their calculation using the finite difference (FD) method. Our *k*_*L*_ predictions using the second order force constant from the FD method (green dot) has also underpredicted the value of *k*_*L*_ by 19% in comparison with the *k*_*L*_ predicted using the second order force constant obtained from DFPT method. Moreover, accurate theoretical predictions enable us to arrive at a rational expression for the porosity correction, as there is a multitude of expressions to choose from, depending on the material and temperature range.

Furthermore, we probe the relative contribution of the acoustic and optical phonon modes to the total *k*_*L*_ of CeO_2_. Typically, for ceramic materials, there is a tendency to neglect the optical contribution owing to their consistently low group velocities and shorter lifetimes^41^. However, the Uranium dioxide (UO_2_) with a reported critical optical mode is an exception^42^. In the wake of this, it would be interesting to know the mode-wise thermal conductivity of the popular nuclear fuel surrogate CeO_2_. To best of author’s knowledge, the mode-dependent *k*_*L*_ of CeO_2_ has not received enough attention. Figure 4b shows the simulated contribution of mode-wise *k*_*L*_ of CeO_2_ compared with UO_2_ at room temperature previously calculated by Pang *et al*.^42^. In CeO_2_ the optical phonons contribute to about 27% of the total *k*_*L*_ compared to the acoustic modes. The optical contributions at 300 K can be precisely broken down to 13.1%, 13.7%, and ~1% from the doubly degenerate transverse optical mode, the triply degenerate Raman active mode and the non-degenerate longitudinal optical mode, respectively. The theory predicts that like UO_2_, its surrogate CeO_2_ will also have strong optical mode contribution to *k*_*L*_. However, in the case of UO_2_, Pang *et al*.^42^ have experimentally proved that the contribution of the TO branch to thermal conductivity is, in fact, higher than the theoretical prediction. Our theoretical predictions suggest an even more significant contribution of the TO branch towards thermal conductivity of CeO_2_, and could, therefore, make an experimental validation worthwhile.

### Effect of porosity on the thermal conductivity using MD simulations and experiments

So far in this manuscript, the primary focus was in understanding the underlying physics of thermal transport and determining the *k*_*L*_ of a defect-free single crystal CeO_2_ using first principles approach. However, manufacturing a defect-free specimen of CeO_2_ with 100% theoretical density (TD) is impracticable, and most importantly *k*_*L*_ of a sample with porosity and defects are expected to be lower than 100% dense CeO_2_. Therefore, it is essential to determine the effect of porosity on *k*_*L*_ of CeO_2_ quantitatively. Hence, the effect of porosity on the *k*_*L*_ of CeO_2_ has been determined using both simulation (MD approach) and experiment. It has to be noted that evaluation of the effect of porosity using first principles is still in genesis, and for that reason, MD simulations were used. Moreover, the theoretical prediction of *k*_*L*_ using both BTE and MD approach complement each other well. The BTE calculates the quantum mechanical scattering rates directly by considering only the lowest order of phonon-phonon interactions and hence become less accurate at very high temperatures where higher order interactions become significant. In contrast, the MD simulations based on classical potential are less reliable at a lower temperature. However, at high temperatures, the phonon-phonon interactions of all orders are duly considered.

In this work, the *k*_*L*_ of CeO_2_ by MD simulations were calculated on systems of 8 × 8 × 8 (6144 atoms), 10 × 10 × 10 (12000 atoms), 12 × 12 × 12 (20736 atoms) and 20 × 20 × 20 (96000 atoms), using the Embedded Atom Many-body (EAM) potentials developed by Cooper *et al*.^43^. Table 2 shows that there is no noticeable difference in the *k*_*L*_ of CeO_2_ at 300 K with different supercell sizes, suggesting that 8 × 8 × 8 system is sufficient enough to represent all the phonon modes available to reproduce the phonon-phonon scattering present in the bulk CeO_2_^44^. For investigating the porosity influence on the *k*_*L*_ of CeO_2,_ two cases with the cell sizes of 10 × 10 × 10 and 20 × 20 × 20 were considered. In each of the supercells, only one pore was introduced by manually removing 5% of atoms, in such a way that for every cerium atom two oxygen atoms are considered, to maintain the charge neutrality of the system. The supercell with vacancies are then made to relax keeping the cell volume same, and then MD calculations were carried out to evaluate the thermal conductivity of porous CeO_2_ (as explained in section 4.1).

**Table 2:** The size dependence of *k*_*L*_ of bulk CeO_2_ at a temperature of 300 K presented using EMD simulation and the Green-Kubo method.

Size of the supercell	Number of atoms	Thermal conductivity (W/mk) at 300 K
8 × 8 × 8	6144	18.87
10 × 10 × 10	12000	18.35
12 × 12 × 12	20736	18.35
20 × 20 × 20	96000	18.84

**Figure 5:**
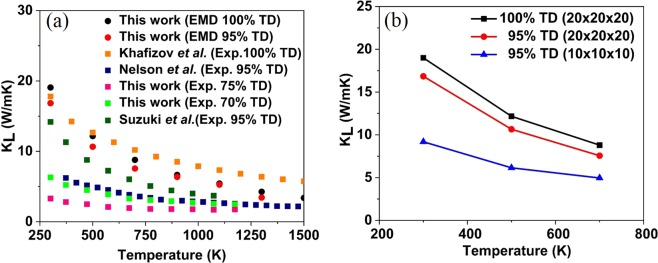
(**a**) Simulated and experimental *k*_*L*_ of porous CeO_2_ as a function of temperature. (**b**) Influence of porosity on the *k*_*L*_ of CeO_2_, by considering the same porosity on a cell size of 10 × 10 × 10 (blue line) and 20 × 20 × 20 (red line) unit cells.

Figure 5a compares the experimental data to the predicted *k*_*L*_ values of CeO_2_ using MD for a pure crystalline CeO_2_ (black dots) and porous CeO_2_ (red dots) modelled on a 20 × 20 × 20 supercell. The *k*_*L*_ values predicted for pure crystalline CeO_2_ are in reasonable agreement with the experimental data (orange square dots) provided by Khafizov *et al*.^8^. As expected, the *k*_*L*_ of the porous CeO_2_ is lower than the pure crystalline CeO_2_. The measurements made by Nelson *et al*.^7^ (navy blue dots) for a specimen of 95% TD was 6.22 W/mK at 100 °C, which is a reduction of almost 55% in the *k*_*L*_ value when compared to the pure crystalline CeO_2_. Suzuki *et al*.^45^ (dark green square dot) investigated the reason for this unusual degradation in the *k*_*L*_ value of a 5% porous CeO_2_ and found that the purity of CeO_2_ is an essential factor (in work done by Nelson *et al*. cerium constituted only 98% of the metallic content of the feedstock). Furthermore, our EMD simulations performed for a 5% porous CeO_2_ (red dots) are in reasonable agreement with the work done by Suzuki *et al*.^45^. Besides, we also noted that *k*_*L*_ values predicted for porous CeO_2_ is significantly dependent on the size of supercell considered. Even though the porosity was modelled the same way in both the supercells, the *k*_*L*_ for the case of 10 × 10 × 10 is considerably lower than the case of 20 × 20 × 20. The Fig. 5b clearly shows that compared to the pure crystalline CeO_2_ at 300 K the *k*_*L*_ predicted for a porous CeO_2_ on a 10 × 10 × 10 supercell has reduced by 52%, whereas the same amount of porosity created on a 20 × 20 × 20 supercell decreased the *k*_*L*_ by 12%. The fact that the *k*_*L*_ is significantly lower for the 10x10x10 case and can be explained by the increased phonon scattering on the surface of the pores due to the decreased average distance between the pores, which directly can be compared to the mean free path of the phonons, as shown previously by Nichenko *et al*.^46^

To validate the theoretical predictions and further expand the quantitative understanding of the effect of porosity on thermal conductivity, the experiments were carried out on the specimens prepared by SPS technique. The pellets of CeO_2_ with varying densities were made by controlling the sintering parameters such as the sintering temperature, pressure, hold time, and the sintering atmosphere. The more detailed description of the sintering conditions and its effect on density and the microstructure of CeO_2_ are given in our previous work^47^. Therefore for brevity, discussions would be limited to the samples on which the thermal conductivity measurements were made. In this work, the experiments were performed on CeO_2_ pellets prepared at a sintering temperature of 1000 °C and 1100 °C by maintaining the pressure and hold time at 50 MPa and 10 min respectively. However, for the benefit of the readers, it is important to note that as the sintering temperature increased (>1500 °C), the CeO_2_ reduced to Ce_2_O_3_. Moreover, in our previous work^47^, it was observed that at a high sintering temperature (>1100 °C) and reductive atmosphere CeO_2_ exhibited a range of stoichiometry and such non-stoichiometric oxide had been susceptible to chemical expansion^48^ leading to the disintegration of the sintered pellets. It was for this reason that the experimental determination of thermal conductivity was limited to two samples sintered at 1000 °C and 1100 °C.

**Figure 6:**
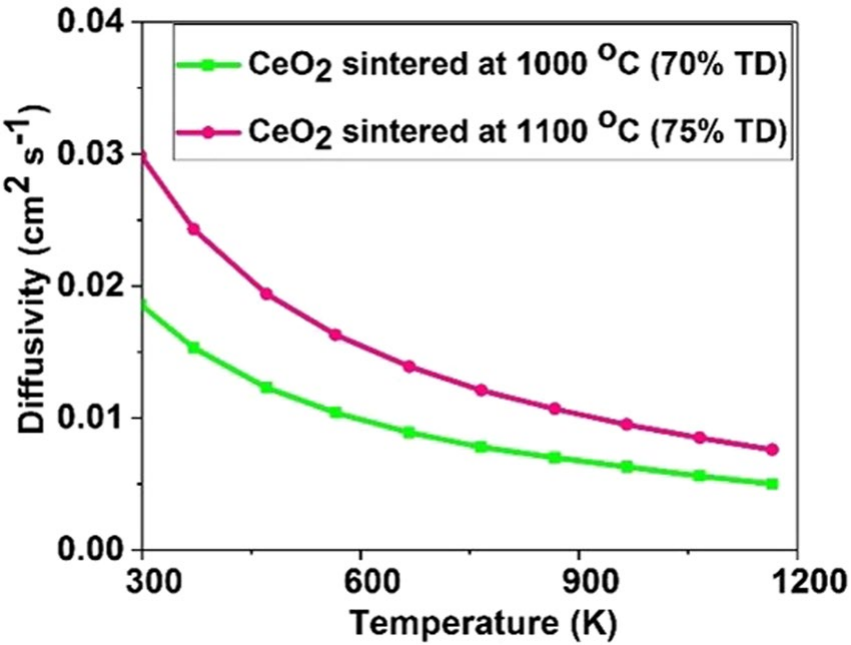
The thermal diffusivity of CeO_2_ as a function of temperature for specimens sintered at 1000 °C and 1100 °C. The uncertainty in the data point from the laser flash apparatus is ±4.5%.

These sintered pellets were characterized using XRD for the determination of the phase, and the XRD patterns revealed that the pellet sintered at 1000 °C (green line) and 1100 °C (pink line) have a face-centred cubic crystal structure (as illustrated in S.I.2 (Fig. S2)). The density of CeO_2_ was measured using the Archimedes principle, and the density of these pellets sintered at 1000 °C and 1100 °C were 70% and 75% respectively. As stated earlier in section 4.2, the thermal diffusivity can be determined using the Laser flash apparatus and Fig. 6 shows the variations of thermal diffusivity of these pellets as a function of temperature. Figures 5a and 6 respectively demonstrate that higher the density of the pellet, higher the thermal diffusivity and thermal conductivity. Moreover, we found that the density of the specimens played a vital role in the measured *k*_*L*_ values (as shown in Fig. 6) at lower temperatures (<1000 °C), however, at a higher temperature, the difference is less significant.

### Nanoscale size effect, cross-plane, and in-plane thermal conductivity

CeO_2_ nanostructures like wires, films, porous and nanocrystalline materials find applications in energy conversion, sensors, and microelectronics where tailoring of thermal transport property is essential. Moreover, a recent work evinces the capabilities of the ultra-thin CeO_2_ for oxygen storage^49^. It is established that the nanostructure surfaces significantly reduce the *k*_*L*_ compared to its bulk counterpart, due to the scattering of the energy carriers. The interplay between the characteristic length and the bulk MFP of the energy carriers is the fundamental physics that determines the dominance of the boundary scattering. The size of the crystalline domain, therefore, acts as limiting length for phonons MFP. Pertaining to this context, a detailed quantitative understanding of the MFP of CeO_2_ is certainly advantageous.

**Figure 7:**
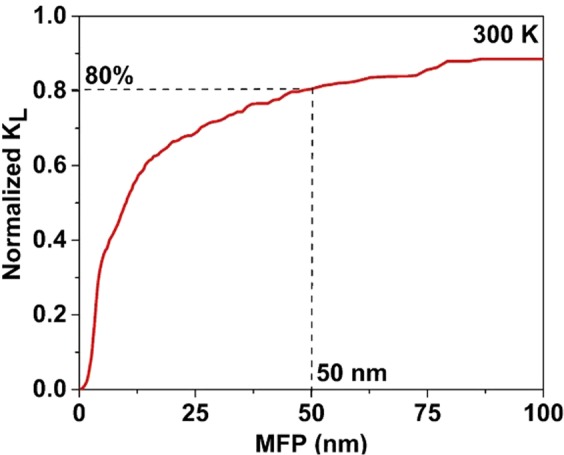
Thermal conductivity accumulation as a function of phonon MFP at room temperature.

In Fig. 7 the dependence of normalized cumulative *k*_*L*_ on phonon MFP at 300 K are presented. The normalized *k*_*L*_ increases with the increase in MFP, with significant contributions to the *k*_*L*_ of CeO_2_ coming from the phonons with MFP between 1 nm to 100 nm. The contribution of phonons mode to *k*_*L*_ is uneven, and the phonons of MFP below 50 nm constitute 80% of *k*_*L*_, indicating that curtailing the size below 50 nm can effectively reduce the *k*_*L*_ of CeO_2_. Therefore, this grasp of the MFP of CeO_2_ will aid the selection of sample size (thickness for thin film, diameter for nanowires and nanoparticles) for diverse technological applications that require a notable deviation from bulk thermal properties.

**Figure 8:**
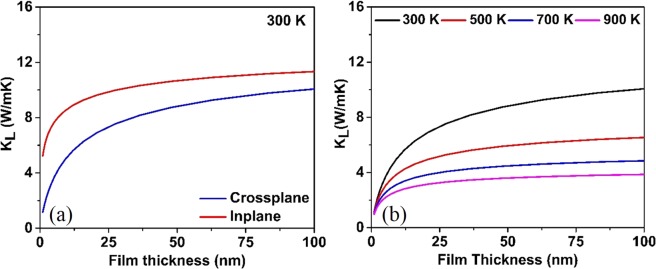
(**a**) In-plane and cross-plane thermal conductivity of CeO_2_. (**b**) Cross-plane conductivity as a function of temperature.

To investigate the influence of nanostructuring on *k*_*L*_ of CeO_2_, we demonstrate the thickness dependence in the cross-plane and in-plane *k*_*L*_ of CeO_2_ along the (001) and (100) planes respectively at 300 K, as shown in Fig. 8a. For these predictions, we utilized almaBTE^50^ a solver of the space-time dependent BTE for phonons in the structured material. The effective in-plane (‖) and cross-plane (⊥) thermal conductivity (*k*_*eff*_) in relaxation time approximation for a film of thickness *L* are evaluated as:

4 $${k}_{eff}(L)=\sum _{q}\,{S}_{q}(L){C}_{q}\parallel {v}_{q}\parallel {\wedge }_{q}\,{\cos }^{2}{\vartheta }_{q}$$

where *S* is, the suppression function that considers the additional phonon scattering instigated by the film boundaries, *C*_*q*_ is the mode contribution to the volumetric heat capacity, *v*_*q*_ the group velocity, ∧_*q*_ is the mean free path and ϑ is the angle between the group velocity and transport axis. The sum over *q* must be interpreted as the combination of a sum of branches and an average over the Brillouin zone. As anticipated, due to the boundary scattering the in-plane and cross-plane *k*_*L*_ of thin films reduced with the reduction in the thickness. If the thickness of CeO_2_ is slashed to 10 nm, its cross-plane (in-plane) *k*_*L*_ is only 4.96 (8.50) W/mK at 300 K. To verify the theoretical findings, we compared the results with experiment. It should be noted that theory predicts the *k*_*L*_ for the infinite two-dimensional CeO_2_ and therefore, the presence of defects would further scatter the phonons and reduce the *k*_*L*_ of CeO_2_ films. Khafizov *et al*.^8^ reported *k*_*L*_ of 7.2 W/mK, for a film of long columnar grain size structure with an average column radius of ~450 nm and the film thickness of 12 μm.

Here, the thickness of 12 μm exceeds the condition that the characteristic size of CeO_2_ must be 50 nm to have a significant reduction in *k*_*L*_. However, our simulation predicts a *k*_*L*_ of 13.0 W/mK for a thickness of 12 μm. The difference between theory and experiment can be primarily associated with the presence of defects and not to the effect of nanostructuring. Therefore, we can surmise that CeO_2_ thin films below ~50 nm with defects can reduce the *k*_*L*_ drastically. Metal oxides generally have large Seebeck coefficient at high temperatures and hence can be considered as candidate materials for advanced thermoelectric.

The coating of CeO_2_ on several alloys has improved the oxidation resistance and can be used in high-temperature applications in industries such as automobiles, aerospace and nuclear, where the knowledge of CeO_2_ cross-plane thermal conductivity as a function of coating thickness and temperature becomes vital. Therefore, in Fig. 8b we have presented the effect of temperature on the *k*_*L*_ of thin film and have observed that the *k*_*L*_ for thin films reduces considerably with an increase in temperature. The presented theoretical work can equip material scientists with vital information required for designing CeO_2_ thin films of optimal thickness that will aid in the design of experiments.

## Summary

We have presented an extensive analysis of the *k*_*L*_ of CeO_2_ both in its bulk and nanoforms. The theoretical predictions of *k*_*L*_ using first principles unified with BTE not only provides an insight into the underlying physics of *k*_*L*_ of CeO_2_ but also helps explain the large discrepancy in the *k*_*L*_ value of CeO_2_ reported previously. The structural and mechanical properties of CeO_2_ could be predicted with better precision by the recently developed pseudopotential with PBEsol functional than any other previously reported DFT calculations. The phonon dispersions spectra of CeO_2_ is evaluated by both direct approach and linear response approach and could describe its polar nature while aptly showing good agreement with the experiment. An investigation of the available three-phonon scattering phase space and mode Grüneisen parameter of CeO_2_, reveals that the lower *k*_*L*_ of CeO_2_ is primarily due to increased scattering and strong anharmonicity respectively. Along with the theoretical investigation of *k*_*L*_ and its dependence on temperature, we also predict the mode wise contribution to the total *k*_*L*_ of CeO_2_. The analysis of mode wise *k*_*L*_ of CeO_2_ indicated the notable contribution to the overall thermal conductivity from the optical modes (~30%), akin to UO_2_. Additionally, we prepared CeO_2_ pellets of varying density by SPS technique and measured the thermal conductivity of these pellets using laser flash technique. The experimental analysis of the samples reveals the dependence of sintering parameters on the density of the CeO_2_ sample and its effect on its measured thermal conductivity. Moreover, we also conducted a theoretical study using the classical MD approach to corroborate the effect of porosity on thermal conductivity. Besides the detailed investigation of the thermal conductivity of CeO_2_ in its bulk form, this article also sheds light on the thermal transport property of nanostructured CeO_2_. We demonstrate the phonon MFP distribution of CeO_2_ is critical in the study of nanostructured materials and devices. The contribution of phonons modes to *k*_*L*_ is uneven, and the phonons of MFP below 65 nm constitute 80% of *k*_*L*_, indicating that limiting the size below 65 nm can efficiently reduce the *k*_*L*_ of CeO_2_. The in-plane and cross-plane thermal conductivity of CeO_2_ thin film as a function of film thickness are also reported. These findings have an impact on heat dissipation in nanoelectronics and photonics, as well as the design of nanostructured thermoelectric materials with reduced thermal conductivity. To conclude, this work serves to moderate the existing ambiguity in the thermal conductivity value of CeO_2_ and provides practical information on CeO_2_ nanostructuring that will potentially meet the demands of numerous industrial applications.

## Methods

### Computational details

All the first principles calculations were performed using the DFT as implemented in the open source Quantum ESPRESSO^51^ code. The pseudopotential used were norm conserved, and the electronic exchange-correlation is based on the generalized gradient approximation (GGA) with the Perdew, Burke, and Ernzerhof functional for solids (PBEsol)^52^. Geometry optimization evaluates the structural properties at zero Kelvin temperature by minimizing the total energy by varying both cell parameter and atom positions. We obtained the total energy convergence of CeO_2_, using an electron wave vector grid and the plane wave energy cutoff of 950 eV and 8 × 8 × 8 respectively. The criteria for the electronic energy convergence and the force convergence was respectively set a value of 10^−12^ eV and 10^−7^ eV/Å. The ground state structural properties were also assessed using the Birch-Murnaghan equation of state^53^. The elastic constants were calculated using the stress-strain method^26,27^. From the elastic constants, the mechanical properties such as the bulk modulus (*B*), shear modulus (*G*), Young’s modulus (*Y*), and Poisson’s ratio (*η*) were determined using the Voigt-Reuss-Hill averaging scheme^30–32^. Because we are analyzing a cubic, polycrystalline structure, therefore the elastic moduli can be evaluated assuming an isotropic aggregate of grains with non-isotropic elastic properties. For a cubic symmetry, there are three independent elastic constants: *C*_11_, *C*_12_, and *C*_44_. The bulk modulus for a cubic structure is the same for the Voigt, Reuss, and Hill averages, as shown in Eq. (5).

5 $$B=\frac{1}{3}({C}_{11}+2{C}_{12})$$

Eq. (6) gives the shear modulus in the Voigt average:

6 $${G}_{V}=\frac{{C}_{11}-{C}_{12}+3{C}_{44}}{5}$$

while Eq. (7) gives the Reuss average:

7 $${G}_{R}=\frac{(5({C}_{11}-{C}_{12}){C}_{44})}{4{C}_{44}+3({C}_{11}-{C}_{12})}$$

The arithmetic mean (Hill) *G* = (*G*_*V*_ + *G*_*R*_)/2 is taken as shear modulus.

The Young’s modulus is calculated as:

8 $$Y=\frac{9BG}{3B+G}$$

The Poisson’s ratio is calculated as:

9 $$\eta =\frac{3B-2G}{2(3B+G)}$$

The *k*_*L*_ of CeO_2_ is predicted by solving the phonon BTE iteratively using the open source ShengBTE^13^ code. The inputs required to solve the BTE are the second order force constants (harmonic force constant) and third order force constants (anharmonic force constants), which are defined as the second and third derivative of the potential energy (*V*) with respect to the atomic displacements respectively. The potential energy of the crystal with a unit cell characterized by a vector ‘*l*’ and the atomic positions in each unit cell described by the vector ‘*b*’, can be expanded in a Taylor series in power of the atomic displacement *u* (*l*, *b*). The first three terms of such a Taylor series expansion is given in Eq. (10) as stated in the ref.^54^.

10 $$\begin{array}{rcl}V & = & {V}_{o}+\,\sum _{lb\alpha }\,\frac{\partial V}{\partial {u}_{\alpha }(l,b)}|\,{}_{0}\,{u}_{\alpha }(lb)+\frac{1}{2}\sum _{lb,l^{\prime} b^{\prime} }\,\sum _{\alpha \beta }\,\frac{{\partial }^{2}V}{\partial {u}_{\alpha }(lb)\partial {u}_{\beta }(l^{\prime} b^{\prime} )}|\,{}_{0}\,{u}_{\alpha }(lb){u}_{\beta }(l^{\prime} b^{\prime} )\\  &  & +\,\frac{1}{3!}\sum _{{\rm{lb}},{\rm{l}}^{\prime} {\rm{b}}^{\prime} ,{\rm{l}}^{\prime\prime} {\rm{b}}^{\prime\prime} }\,\sum _{{\rm{\alpha }}{\rm{\beta }}{\rm{\gamma }}}\,\frac{{\partial }^{3}{\rm{V}}}{\partial {{\rm{u}}}_{{\rm{\alpha }}}({\rm{lb}})\partial {{\rm{u}}}_{{\rm{\beta }}}(l^{\prime} b^{\prime} )\,\partial {{\rm{u}}}_{{\rm{\gamma }}}1^{\prime\prime} {\rm{b}}^{\prime\prime} )}|\,{}_{0}\,{{\rm{u}}}_{{\rm{\alpha }}}({\rm{lb}}){{\rm{u}}}_{{\rm{\beta }}}(l^{\prime} b^{\prime} ){{\rm{u}}}_{{\rm{\gamma }}}({\rm{l}}^{\prime\prime} {\rm{b}}^{\prime\prime} )\end{array}$$

The second order force constant (${{\rm{\Phi }}}_{{\rm{\alpha }}{\rm{\beta }}}(lb,l^{\prime} b^{\prime} )$) and the third order force constant (${{\rm{\Phi }}}_{{\rm{\alpha }}{\rm{\beta }}{\rm{\gamma }}}({\rm{lb}},{\rm{l}}^{\prime} {\rm{b}}^{\prime} ,{\rm{l}}^{\prime\prime} {\rm{b}}^{\prime\prime} )$ are defined as shown in Eqs (11) and (12) respectively.

11 $${\Phi }_{\alpha \beta }(lb,l^{\prime} b^{\prime} )={\frac{{\partial }^{2}V}{\partial {u}_{\alpha }(lb)\partial {u}_{\beta }(l^{\prime} b^{\prime} )}|}_{0}$$

12 $${\Phi }_{\alpha \beta \gamma }(lb,l^{\prime} b^{\prime} ,l^{\prime\prime} b^{\prime\prime} )={\frac{{\partial }^{3}V}{\partial {u}_{\alpha }(lb)\partial {u}_{\beta }(l^{\prime} b^{\prime} )\partial {u}_{\gamma }(l^{\prime\prime} b^{\prime\prime} )}|}_{0}$$

**Figure 9:**
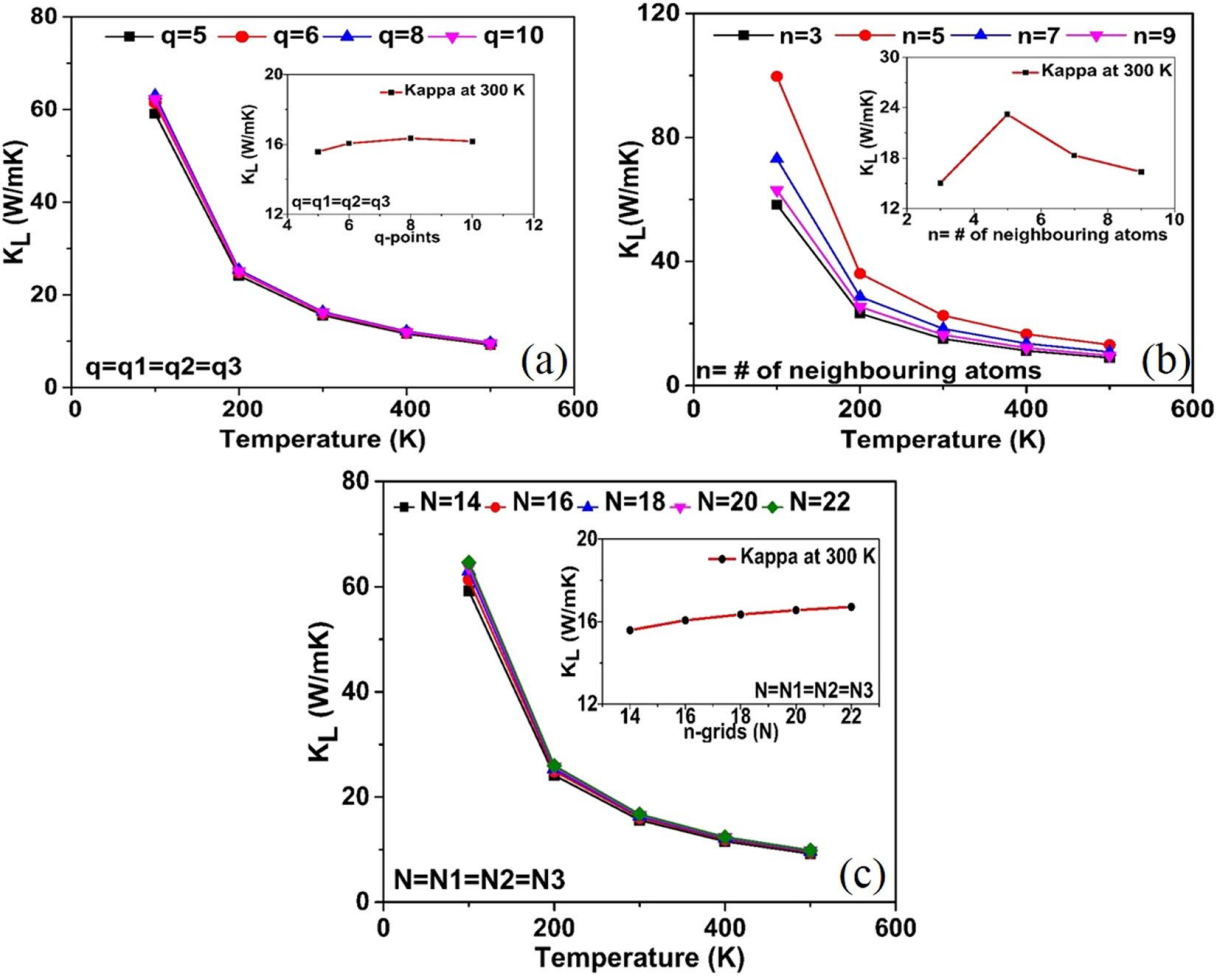
Convergence of the *k*_*L*_ with respect to (**a**) the q-points considered for the second order force constants (by maintaining n = 9 and N = 18 × 18 × 18 for all the cases); (**b**) the number of neighboring atoms considered for calculating the third order force constant (by keeping q = 8 × 8 × 8 and N = 18 × 18 × 18 for all the cases); (**c**) the number of grid planes along each axis in reciprocal space for solving the BTE (by considering q = 8 × 8 × 8 and n = 9 for all the cases).

The dynamical matrices for evaluating the phonon density of states is obtained from the second order force constant as follows,

13 $${D}_{\alpha \beta }(bb^{\prime} {|}_{q})=\frac{1}{\sqrt{{m}_{b}{m}_{b^{\prime} }\,}}\,\sum _{{l}^{\text{'}}}\,{{\rm{\Phi }}}_{\alpha \beta }(0b,l^{\prime} b^{\prime} )exp(iq.l^{\prime} )$$

where *q* is the wave vector and *m* denote the mass of an atom at a site in the crystal. Further, diagonalizing the dynamical matrix yields the phonon frequencies (ω), which in turn provides the phonon group velocities and heat capacity at a fixed volume (*C*_*v*_). In this work, the harmonic force constant is calculated using both linear approach (DFPT)^12^ and direct approach (Parlinski-Li-Kawazoe method)^35^, based on the supercell approach with finite displacement method as implemented in the Phonopy package^55^. To obtain the converged phonon properties, the calculations of the harmonic force constant were done using a *q*-point mesh of 8 × 8 × 8 (phonon dispersion for different q-points are shown as S.I.3, (Fig. S3)) and a 5 × 5 × 5 supercell of the primitive cell containing 375 atoms. The calculation of the third order force constants was performed on a 4 × 4 × 4 supercell, and the force cutoff distance was set to the ninth nearest neighboring atoms. The convergence of the *k*_*L*_ with respect to the number of q points used in the second order force constant, the number of neighboring atoms considered in third order force constant and the number of grid planes along each axis in the reciprocal space for solving the BTE is detailed in Fig. 9. From the cubic force constants, the phonon scattering processes are evaluated using Fermi’s golden rule, and finally, the *k*_*L*_ is calculated using the iterative solutions of the BTE as implemented in ShengBTE. In the approach implemented in ShengBTE, Eq. (14) gives the expression to compute the lattice thermal conductivity tensor,

14 $${k}_{L}^{\alpha \beta }=\frac{1}{N{K}_{B}{T}^{2}{\rm{\Omega }}}\,\sum _{{\rm{\lambda }}}^{\infty }\,{f}_{0}({\omega }_{{\rm{\lambda }}}){f}_{0}({\omega }_{{\rm{\lambda }}}+1){({\rm{\hbar }}{\omega }_{{\rm{\lambda }}})}^{2}{v}_{{\rm{\lambda }}}^{\alpha }{v}_{{\rm{\lambda }}}^{\beta }{\tau }_{{\rm{\lambda }}}$$

where Ω is the volume of the primitive cell, *α* and *β* are the Cartesian components of *x*, *y*, or *z*, *k*_B_ is the Boltzmann constant, *ω*_*λ*_ and *υ*_*λ*_ are the angular frequency and group velocity respectively, *τ*_*λ*_ is the relaxation time of mode λ and *f*_0_(*ω*_λ_) is the Bose-Einstein distribution function. The *k*_*L*_ presented in this work are the fully iterative solution of the Peierls-equation, and the convergence of *k*_*L*_ with the number of iterations starting from the zeroth-order approximation (which is equivalent to operating under RTA) and the *k*_*L*_ at low temperature (<300 K) are shown in S.I.4 (Fig. S4) and S.I.5 (Fig. S5) respectively.

For a polar material, the interatomic forces are divided into two additive contributions; the analytic and non-analytic contributions (nac). The analytic contribution accounts for all the forces under the restricted periodic boundary conditions under which the averaged electric field is assumed to be zero. The nonanalytic contribution accounts the additional forces owing to non-zero averaged electric field^56^. The classical Newton’s second law of motion for describing the atomic vibrations for a polar solid is as shown in Eq. (15),

15 $$mj({\partial }^{2}\frac{{u}_{\alpha }(t,j;P)}{\partial {t}^{2}})=-\,(\frac{\partial E(U)}{\partial {u}_{\alpha }(t,j;P)})+e{Z}_{\alpha }(j).E.$$

where *m*_*j*_ denotes the atomic mass of the *j*^*th*^ atom in the primitive unit cell, α (α = x, y, z) is the component of the atomic displacement from its equilibrium position of the *j*^*th*^ atom in the *P*^*th*^ reference primitive unit cell within a supercell, as detailed in ref.^56^. The first term on the right-hand side of the Eq. (15) corresponds to the analytical force arising due to the short-range interatomic interactions. The *U* and *E* (*U*) in the first term represent the whole set of atomic displacements and the total energy respectively. In the second term, the dot product between the Born effective charge (*Z*) and the average of the electric field (*E*) induced by the atomic vibrations accounts for the non-analytic force due to the long-range coulombic interaction. In this work, CeO_2_ being a polar material, the non-analytical contribution was considered, and the Born charges and the dielectric constant required to evaluate the non-analytical contribution were calculated using the DFPT. Therefore, the nac contribution needs to be included in the *k*_*L*_ calculation of CeO_2_.

**Figure 10:**
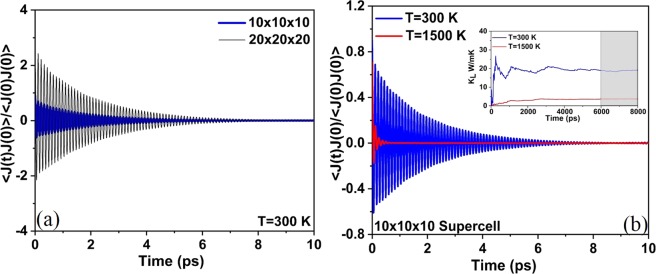
(**a**) The HFACF for CeO_2_ at a temperature of 300 K with a system size of 10 × 10 × 10 and 20 × 20 × 20 unit cells. (**b**) The HFACF for CeO_2_ at a temperature of 300 K and 1500 K with a system size of 10 × 10 × 10 unit cells; inset shows the *k*_*L*_ fluctuations with time and the shaded region indicates the time range over which the HCACF integral is averaged to predict the *k*_*L*_.

For MD simulations, we used the equilibrium classical MD techniques together with the Green-Kubo linear response formalism^57^ as implemented in LAMMPS (Large-scale Atomic/Molecular Massively Parallel Simulator) MD simulation code^58^. The Green-Kubo formalism uses the heat current autocorrelation function (HCACF) (shown in Fig. 10) which decay along a direction as described in ref.^57^. To predict the *k*_*L*_ of CeO_2_, the Verlet leapfrog algorithm was implemented^59^. Further, the system was first simulated in a constant number of atoms, pressure, and temperature (NPT) ensemble for 4 ns to ensure it reached equilibrium at the desired temperatures, then the ensemble was switched into a constant number of atoms, volume, and temperature (NVT) ensemble and ran for 4 ns. The heat current autocorrelation function (HCACF) were estimated along with an NVE ensemble calculation which generates an 8 ns raw heat current data at every calculation. Finally, the *k*_*L*_ value was computed by averaging the *k*_*L*_ over the time range were the fluctuations were minimal as shown in the inset of Fig. 10b.

Finally, to study the effect of the nanostructuring and calculate the in-plane and cross-plane thermal conductivity of thin films of CeO_2_ we used the open source almaBTE code^50^. Recently, an exact solution to evaluate the BTE in the cross-plane geometries has been obtained. The film conductivity at thickness L as an integral of phonon frequency is given as,

16 $$k(L)=\int S(\omega ,L)k(\omega )d\omega $$

where *S* is a suppression function that contains the thin film physics and *k*(ω) denotes the bulk spectral conductivity. More detailed description of the procedure can be found in the following refs^50,60^. It has to be noted that the almaBTE code uses the second order force constant predicted by the FD method. Hence the *k*_*L*_ values for the nanostructured cases are underpredicted by ~19% as shown for the bulk CeO_2_.

### Materials and experimental details

**Figure 11:**
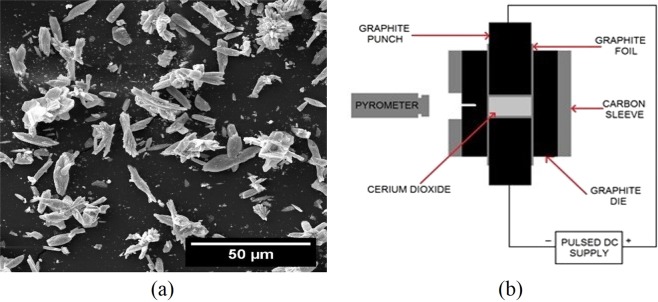
(**a**) Scanning electron micrograph of as-received CeO_2_ powders from ACROS Organic. (**b**) Schematic diagram of SPS sintering of CeO_2_.

The 99.9% pure CeO_2_ powder was obtained from ACROS Organics. The as-received powder was observed under the scanning electron microscope, the micrograph as shown in Fig. 11a revealed that the particles were needle-shaped with dimensions around 20 μm long and 5 μm wide. The as-received powder was sintered using SPS (Thermal Technology LLC 10-3 system) in a graphite die-punch setup as shown in Fig. 11b. The powder die contacts were separated by graphite foils to protect the die from contamination and to reduce friction between the powders and die. A 6 mm hole was drilled from the inner surface of the die, and a pyrometer was focused on this hole to record the temperature of the die. The temperature was controlled by regulating the current passing through the die-punch system, and a Data Acquisition System was used to record displacement, temperature and pressure data.

The pellets were sintered by varying the sintering parameters such as temperature, pressure and hold time. The sintered pellets were then subjected to X-Ray Diffraction (XRD) using BRUKER D8 with Chromium K-alpha radiation to determine the phase and the presence of any residual carbon during the SPS sintering. The Archimedes’ method was used to determine the density of each pellet. Finally, the thermal conductivity was calculated using the laser flash apparatus; Laser flash technique records the thermal diffusivity (*α*) of the specimens using the Parkers relations^61^ given as shown in Eq. (17):

17 $$\alpha =0.1388\times \frac{{L}^{2}}{{t}_{\frac{1}{2}}}$$

where *L* is the thickness of the specimen and t_1/2_ is the half of the maximum time taken for the signal to reach the detector. From the measured *α*, the thermal conductivity as a function of temperature (*k* (*T*)) can be measured using the relation (18):

18 $$\alpha (T)=\frac{k(T)}{\rho (T)\times {C}_{p}(T)}$$

where *ρ*(*T*) and *Cp*(*T*) is the density and heat capacity at constant pressure as a function of temperature respectively. In this work the *Cp* changes as a function of temperature was determined by comparing the maximum value of the temperature rise with that of a reference pellet, using the relation *Cp* = *Q*/(*dT*.*m*), where *Q* represent the energy of the pulsed laser beam, *m* mass of the specimen, and *dT* is the maximum value of the temperature rise. The reference pellet used was a certified stainless steel. However, the density changes as a function of temperature has been kept constant. The thermal conductivity measurements were made on cylindrical pellets of diameter 12.7 mm and thickness 2–3 mm.

## Supplementary information

Atomistic and experimental study on thermal conductivity of bulk and porous cerium dioxide
