# Supplementary material for: Atomistic and experimental study on thermal conductivity of bulk and porous cerium dioxide
Source: Sci Rep. 2019 Apr 19;9:6326. doi: 10.1038/s41598-019-42807-5 (PMC6474893; doi:10.1038/s41598-019-42807-5)
Supplement: Supplementary file 1 — Atomistic and experimental study on thermal conductivity of bulk and porous cerium dioxide [file 41598_2019_42807_MOESM1_ESM.docx]

**Supplementary Informations**

**Atomistic and experimental study on thermal conductivity of bulk and porous cerium dioxide**

**Linu Malakkal^1^, Anil Prasad^2^, Dotun Oladimeji^3^, Ericmoore Jossou^1^, Jayangani Ranasinghe^3^, Barbara Szpunar^3^, Lukas Bichler^2^, Jerzy Szpunar^1^**

^1^Department of Mechanical Engineering, University of Saskatchewan, Canada

^2^School of Engineering University of British Columbia-Okanagan Kelowna, Canada

^3^Department of Physics and Engineering Physics, University of Saskatchewan, Canada

**S.I. 1) Mode Grüneisen coefficient for different cases.**

Fig. S1 (a) shows the changes in mode Grüneisen coefficient (γ_qj_) with different q-points considered while maintaining the other parameters such as the nearest neighbour atoms (n) and the number of grid planes along each axis (N) constant. Similarly, Fig. S1 (b) depicts the variations in γ_qj_ with different neighbouring atoms considered in the third order force constant calculation keeping the q-points and the N-grid constant. Fig. S1(a) indicates that the variations in γ_qj_ with respect to different q-points are not significant whereas, the selection of the number of nearest neighbouring atoms in the third order force constant has a considerable effect on the γ_qj_ of CeO_2_.

| **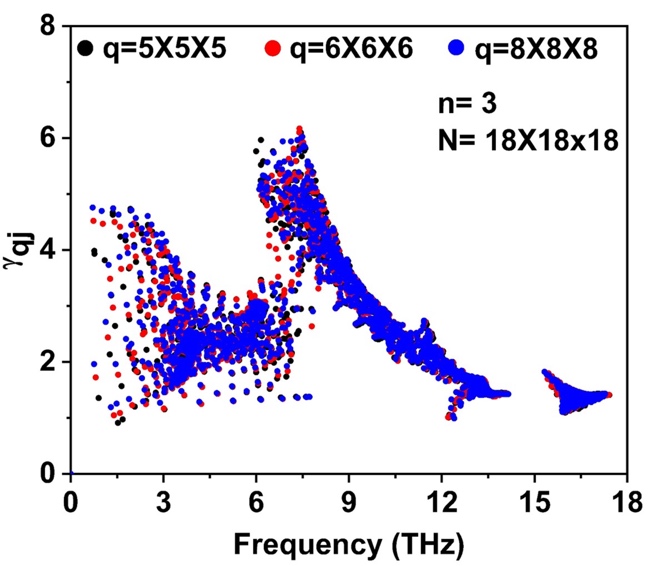** | **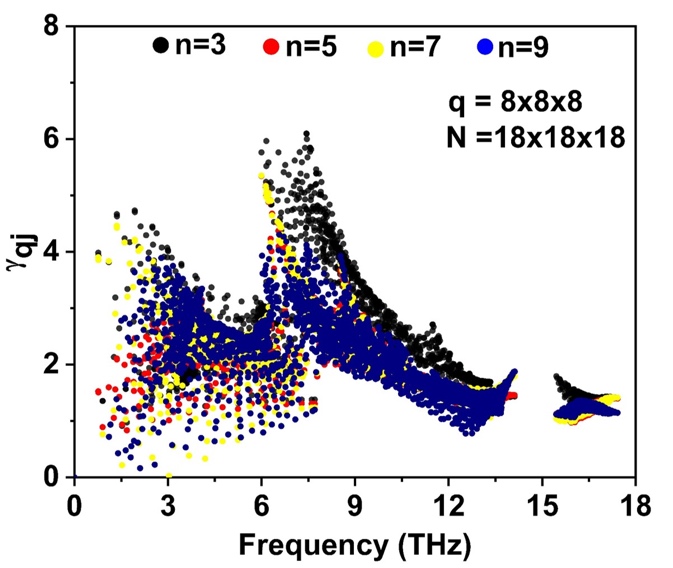** |
| --- | --- |
| (a) | (b) |
| **Figure S1.** Mode Grüneisen coefficient ($\gamma_{qj})$ (a) for different q points (b) for different nearest neighbours. | |

**S.I. 2) XRD peaks of CeO_2_ sintered at 1000 ^o^C and 1100 ^o^C**

| 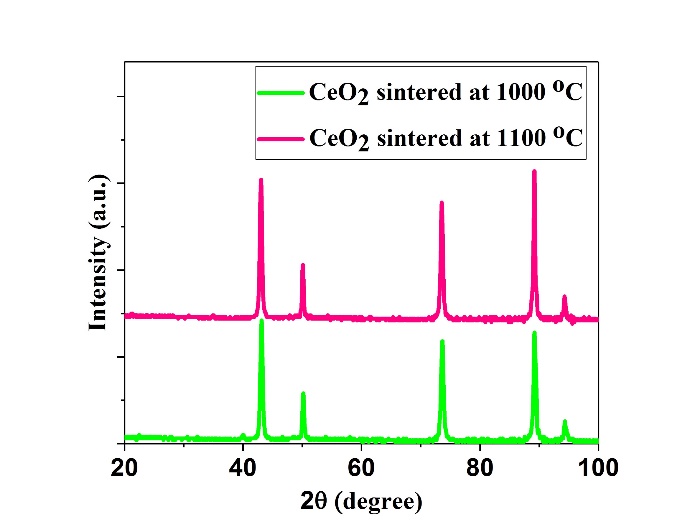 |
| --- |
| **Figure S2.**  XRD peaks of CeO_2_ sintered at 1000 ^o^C and 1100 ^o^C. |

**S.I. 3) Phonon dispersion curves of CeO_2_ for different qpoints.**

| **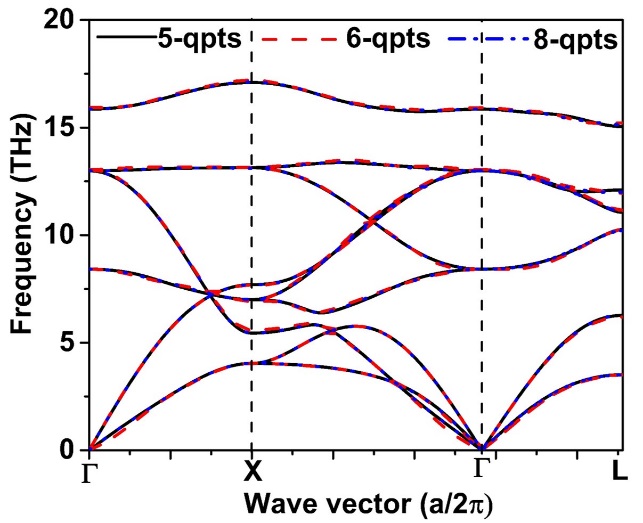** |
| --- |
| **Figure S3.** Phonon dispersion with different q-points from DFPT. |

**S.I. 4) The convergence of *k_L_* with the number of iterations at 300 K.**

| 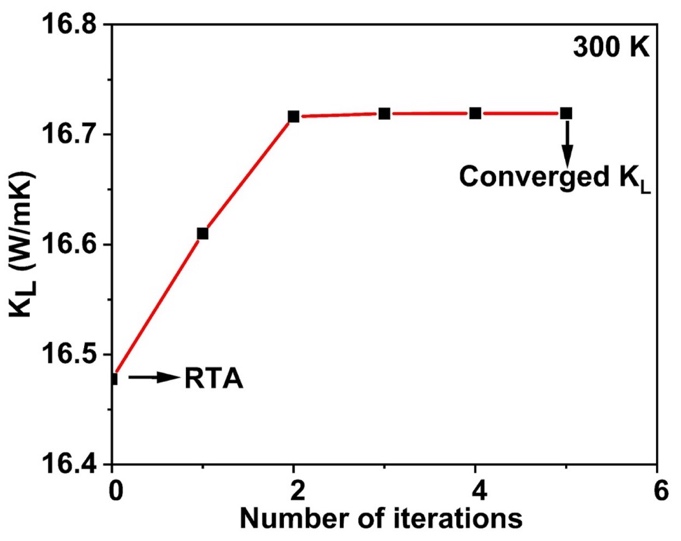 |
| --- |
| **Figure S4.** The convergence of *k_L_* with number of iterations. |

**S.I.5) Low temperature *k_L_*** **of CeO_2_.**

| 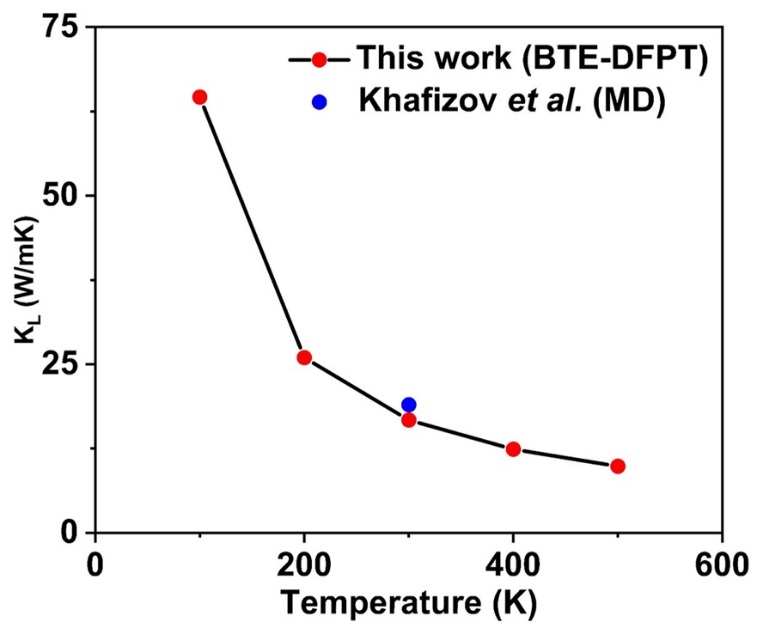 |  |
| --- | --- |
| **Figure S5.** The *k_L_* of CeO_2_ at temperature lower than room temperature compared with the value predicted by Khafizov *et al.*^8^ at 300 K using molecular dynamics (MD) calculations. | |
